# Glutamatergic Mechanisms in Glioblastoma and Tumor-Associated Epilepsy

**DOI:** 10.3390/cells10051226

**Published:** 2021-05-17

**Authors:** Falko Lange, Max Frederik Hörnschemeyer, Timo Kirschstein

**Affiliations:** 1 Oscar-Langendorff-Institute of Physiology, Rostock University Medical Center, 18057 Rostock, Germany; max.f.hmr@gmail.com; 2 Center for Transdisciplinary Neurosciences Rostock, University of Rostock, 18147 Rostock, Germany

**Keywords:** glutamate, ionotropic glutamate receptor, metabotropic glutamate receptor, glioblastoma, epilepsy, seizures, perampanel, preclinical model

## Abstract

The progression of glioblastomas is associated with a variety of neurological impairments, such as tumor-related epileptic seizures. Seizures are not only a common comorbidity of glioblastoma but often an initial clinical symptom of this cancer entity. Both, glioblastoma and tumor-associated epilepsy are closely linked to one another through several pathophysiological mechanisms, with the neurotransmitter glutamate playing a key role. Glutamate interacts with its ionotropic and metabotropic receptors to promote both tumor progression and excitotoxicity. In this review, based on its physiological functions, our current understanding of glutamate receptors and glutamatergic signaling will be discussed in detail. Furthermore, preclinical models to study glutamatergic interactions between glioma cells and the tumor-surrounding microenvironment will be presented. Finally, current studies addressing glutamate receptors in glioma and tumor-related epilepsy will be highlighted and future approaches to interfere with the glutamatergic network are discussed.

## 1. Introduction

Glioblastomas (WHO grade Ⅳ gliomas) represent the most common tumors of the central nervous system, and with an overall 5-year survival of 6.8%, this tumor disease has one of the worst prognoses in the entire oncological spectrum [1]. The median age of onset is 64 years, with an increase at the age of 75–84 years [1]. Primary glioblastomas arise de novo, whereas secondary glioblastomas derive from less-malignant precursor lesions [2]. The majority of glioblastomas is defined as primary, mostly with wild-type isocitrate dehydrogenase 1 (IDH1), whereas most of the secondary cases harbor mutations in IDH1 [3]. Mutations in IDH1 are associated with an increased patient survival [4].

Depending on the genetic and epigenetic alterations [5], four subtypes can be defined [6]. Genes encoding members of the signaling pathways phosphatidylinositol-3-kinase (PI3K) or mitogen-activated protein kinase (MAPK) and the corresponding receptor tyrosine kinases-like epidermal growth factor receptor (EGFR) or platelet-derived growth factor receptor A (PDGFRA) are altered in up to 90% of cases [7,8]. Among the tumor suppressors, loss of function of phosphatase and tensin homolog (PTEN) is the most frequent mutation (30–40%) [6,9]. In approximately 10% of cases, neurofibromin 1 (NF1) is mutated or deregulated [8,9]. With respect to cell cycle control, loss-of-function mutations of cyclin dependent kinase inhibitor 2A/B (CDKN2A/B) can be found in half of all glioblastoma specimens, and cyclin-dependent kinase 4 (CDK4) is often highly amplified. Furthermore, like in other major cancer types [10], tumor protein P53 (TP53) is also subject to mutations.

Despite the knowledge gained on tumorigenesis and the progression of glioblastomas in the last decades, these have so far only insufficiently contributed to improving treatment options. Standard therapy includes resection of the tumor mass followed by adjuvant irradiation and chemotherapy with the alkylating drug temozolomide (TMZ) [11]. This regimen prolongs the median overall survival to 15–18 months (versus 3–4 months without treatment [12]) [11,13,14]. The status of O^6^-methylguanine-DNA methyltransferase (MGMT) promotor methylation is an established prognostic marker of the treatment with TMZ [13,15,16,17], albeit in a Spanish study including more than 300 patients, contrary to previous studies, no beneficial effect of MGMT promotor methylation status could be detected [18]. Combination of TMZ with lomustine, also an alkylating agent, may prolong the survival of patients with methylated MGMT promotors [19]. However, the findings of Herrlinger et al. in 2019 were based on a relatively small cohort (129 patients), and survival successes came at the price of additional side effects. Another add-on therapy is tumor-treating fields that mediate antimitotic effects by alternating electric fields, which can prolong the survival of patients with glioblastoma [20,21]. However, the low level of acceptance of this method has limited its widespread use [22,23].

Glioblastomas are associated with a variety of neurological symptoms and signs including headaches, changes in cognition and personality, gait imbalance, visual deficits and tumor-associated epilepsy [12]. The occurrence of seizures is not only a frequent comorbidity in the course of glioma progression [24] but is often the initial manifestation of the tumor disease [25]. In low-grade gliomas, 70–90% of the patients suffer from seizures at the time of diagnosis, whereas in glioblastoma, seizures appear to be less frequent (up to 60%) [25,26]. Epileptogenesis in the peritumoral tissue is a multifactorial process [27]. Based on numerous preclinical, but also some clinical, studies, there is increasing evidence that the neurotransmitter glutamate plays an important role in the development of seizures and also in the progression of the tumor disease.

Herein, we review our current understanding of the glutamatergic processes at the molecular level that drive glioma progression and presentation of an epileptic phenotype. Based on this, open research questions are presented that could serve to reach a better understanding of the disease in the future.

## 2. Glutamatergic Mechanisms of Glioma Progression and Tumor-Associated Epilepsy

**Figure 1:**
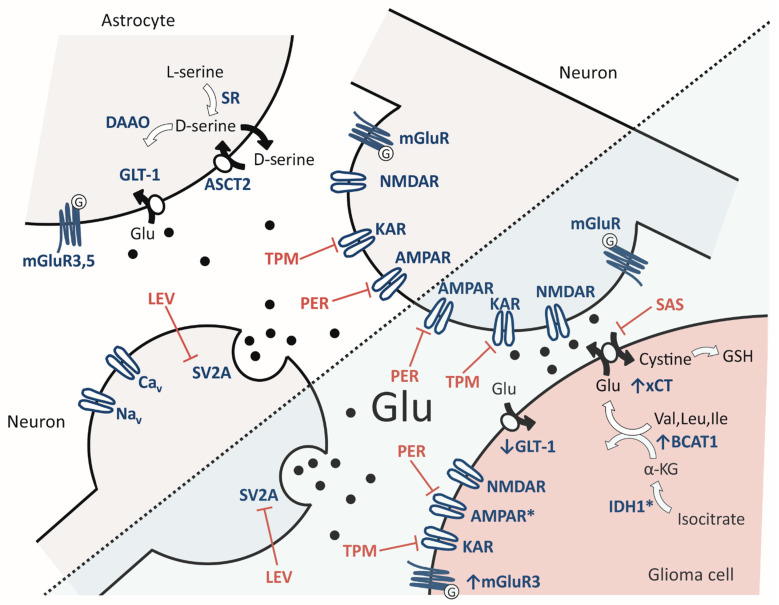
Glutamatergic interaction in health and glioma. Details of the molecular processes are described in Section 2. AMPA receptor (AMPAR), alanine-serine-cysteine amino acid transporter-2 (ASCT2), branched-chain amino acid transaminase 1 (BCAT1), D-amino acid oxidase (DAAO), glutamate transporter 1 (GLT-1), isocitrate dehydrogenase 1 (IDH1), kainate receptor (KAR), levetiracetam (LEV), mGluR (metabotropic glutamate receptor), NMDA receptor (NMDAR), perampanel (PER), sulfasalazine (SAS), serine racemase (SR), synaptic vesicle glycoprotein 2A (SV2A), topiramate (TPM), cystine/glutamate antiporter solute carrier family 7 member 11 (SLC7A11 or xCT).

Glioblastoma and tumor-associated epilepsy share pathophysiological mechanisms that drive both tumor progression and the generation of seizures [28]. One major pathological mechanism is aberrant glutamate signaling within the tumor tissue and its microenvironment. In the glioma-surrounding tissue, extracellular glutamate levels were found to be elevated up to 100-times higher than in unaffected brains [29,30]. On the one hand, high levels of glutamate stimulate proliferation and invasion of glioma cells [31,32,33] and, on the other hand, may lead to epileptic discharges, excitotoxicity and therefore allow for tumor bulk expansion (Figure 1) [34,35].

Primarily, glutamate is released by glioblastoma cells via cystine/glutamate antiporter solute carrier family 7 member 11 (SLC7A11 or xCT) [36]. To counteract oxidative stress, cystine, an essential precursor for glutathione synthesis, is imported in exchange for glutamate. In glioblastoma cells, expression of xCT is often upregulated [37,38,39,40]. This may be in part due to hypoxic conditions in fast-growing glioblastomas [41]. It was reported that the expression levels of xCT were found to be associated with seizures [42,43] and identified as an independent biomarker for glioma-associated seizures [43], also in a cohort of IDH1-wildtype glioblastoma patients [44]. With respect to glioblastoma, no correlation with survival was found [42,43].

Furthermore, the re-uptake of glutamate from the extracellular space is also impaired in glioma tissue, as expression of glutamate transporter 1 (GLT-1), also known as excitatory amino acid transporter 2 (EAAT2), is downregulated, or carriers are mislocalized, and therefore sodium-dependent re-uptake of glutamate is reduced [37,45,46]. The situation may be exacerbated by an overexpression of branched-chain amino acid transaminase 1 (BCAT1) [47]. Again, upregulation may in part be driven by hypoxic conditions, in which HIF-1α was identified as an essential transcription factor [48]. BCAT1 transfers an α-amino group from branched-chain amino acids to α-ketoglutarate, thereby producing glutamate and the respective branched-chain α-ketoacid. This mechanism may contribute to an elevated glutamate level within the cytoplasm of the tumor cells and subsequently an increased glutamate release [47]. In glioblastomas, high expression of BCAT1 was associated with poor survival [49,50].

Isocitrate dehydrogenase 1 (IDH1) mutations are common in low-grade gliomas (>80%) and secondary glioblastomas (73%) but are rare in primary glioblastomas (3.7%) [3]. Instead of catalysis of isocitrate to α-ketoglutarate in the Krebs cycle, in IDH1-mutant glioma cells, D-2-hydroxyglutarate (D-2HG) is generated [51]. D-2HG shares a steric analogy with glutamate and therefore may act as an additional glutamate receptor agonist. Interestingly, D-2HG also affects BCAT1, possibly due to direct inhibition of enzyme activity [52] or DNA hypermethylation within the main promoter region of BCAT1 in IDH1-mutant gliomas [47]. Furthermore, McBrayer et al. showed that in IDH1-mutant gliomas, an additional inhibition of glutaminase (hydrolyzes glutamine to glutamate) by glutaminase inhibitor CB-839 led to an increased cell death under hypoxic conditions in vitro and increased susceptibility to irradiation in vivo [52]. The facilitatory role of IDH1 mutations in epileptogenesis has also been shown in patient-derived samples [53,54].

In terms of the surrounding neurons, excitotoxicity via glutamate-driven hyperexcitation contributes to tumor growth [55]. High levels of this neurotransmitter lead to receptor-mediated Na^+^/Ca^2+^-dependent depolarization that eventually end up in abnormally high levels of intracellular Ca^2+^, which in turn may trigger key players contributing to cell death [56]. At the same time, high levels of extracellular glutamate may attenuate cystine uptake via xCT, thereby cutting off the tumor cells from cysteine as a precursor for the synthesis of the antioxidant glutathione and hence contribute to cellular damage by reactive oxygen species [55]. In addition to glutamate, GABAergic mechanisms with altered intracellular chloride concentration [57] and microglia dysfunction [58] were also described as relevant contributors to epileptogenicity in the tumor-surrounding tissue. Hence, the epileptic phenotype has a multifactorial pathogenesis, and here we will focus on glutamatergic mechanisms.

Glutamate in the synaptic cleft may activate glutamate receptors on tumor cells themselves in an autocrine manner or on proximate neurons and astrocytes in a paracrine manner. The role of ionotropic and metabotropic glutamate receptors will be discussed separately in the following sections.

### 2.1. Ionotropic Glutamate Receptors

**Figure 2:**
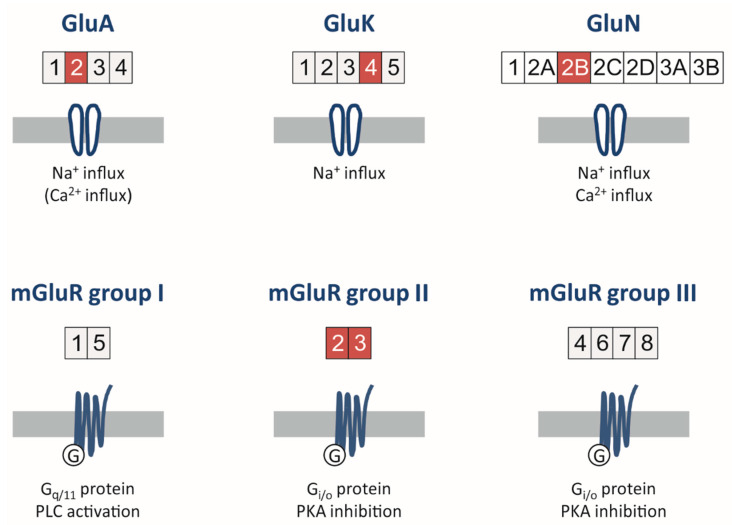
Synopsis of ionotropic (upper row) and metabotropic (lower row) glutamate receptors. Subunits detected in glioblastomas are highlighted in red.

Glutamate receptors are subdivided into ionotropic and metabotropic receptors (Figure 2). Ionotropic receptors have been named after the prototypical glutamate agonists α-amino-3-hydroxy-5-methyl-4-isoxazolepropionic acid (AMPA receptors), kainic acid (kainate receptors) and N-methyl-D-aspartate (NMDA receptors).

#### 2.1.1. AMPA Receptors

AMPA receptors (AMPARs) are tetrameric complexes composed of GluA1, GluA2, GluA3, or GluA4 subunits in various combinations and predominantly responsible for fast excitatory transmission in the central nervous system [59]. All subunits, albeit GluA1 to a lesser extent, have been detected in glioblastoma cell lines and patient-derived specimens [60,61]. Whereas immature neurons express low levels of GluA2-containing AMPARs, which confers Ca^2+^-permeability and an inwardly rectifying current–voltage relationship, GluA2 expression increases during postnatal development [62,63]. At adult synapses, however, GluA2 subunits typically show full adenosine-to-inosine mRNA editing by virtue of the enzyme ADAR (adenosine deaminase acting on RNA) at the Q/R-site leading to a translational modification from glutamine (Q) to arginine (R) [64]. In contrast, unedited GluA2 subunits also form Ca^2+^-permeable AMPARs and seem to be frequently expressed in glioma tissues [61,65,66,67], and importantly, these receptors appear to promote proliferation and migration [68]. Since the discovery of glutamatergic synapses between neurons and glioma cells (Section 2.3), Ca^2+^-permeable AMPARs have been regarded as attractive candidates for specific antagonists to interfere with the tumor-promoting effects of glutamate [67].

Since the non-competitive AMPAR antagonist, perampanel (PER), has been developed to treat patients with partial and generalized seizures [69,70], this compound has attracted much interest in treating glioma-associated seizures due to the hypothesis that glutamate released from glioma cells could not only activate surrounding neurons to cause seizures and lead to excitotoxicity but could also promote glioma progression [67]. Along these lines, PER could be effective in both seizure control and tumor repression (Section 2.4).

#### 2.1.2. Kainate Receptors

Kainate receptors (KARs) are tetrameric transmembrane proteins composed of three low-affinity subunits, GluK1 to GluK3 (glutamate receptor, kainate-subtype, formerly named GluR5 to GluR7), and two high-affinity subunits, GluK4 (KA1) and GluK5 (KA2), in homo- and heteromeric combinations [71,72]. Our notion on the expression of KARs in gliomas is scarce, but at least in pediatric glioblastoma, GluK3 and GluK4 were found to be expressed at levels higher than in the human brain, whereas low-grade astrocytomas only showed increased expression of GluK4 [61]. This is consistent with the high expression of GluK4 in U373 glioma cells [73] and U87MG cells [60] as well as in HAP-1 glial precursor cells [74]. However, the role of KAR expression on glioma cells in tumor progression needs to be established, since glutamate released from glioma cells might activate KARs along with AMPARs in an autocrine fashion [32]. On the other hand, neuronal KAR activation by astrocyte-released glutamate can be highly specific, since glutamatergic effects on interneurons in the CA1 stratum radiatum were found to be confined to GluK1-containing KARs [75]. Using the highly selective GluK1-inhibitor topiramate (TPM) [76] or establishing novel subunit-specific KAR inhibitors such as piperazine-2,3-dicarboxylic acid derivatives [77], future studies are needed to focus on the role of KARs in glioma progression and glioma-associated epilepsy.

#### 2.1.3. NMDA Receptors

NMDA receptors are tetrameric transmembrane proteins composed of two glycine/D-serine-binding GluN1 (glutamate receptor, NMDA-subtype) subunits and two glutamate-binding GluN2 or GluN3 subunits [59,78]. While there is only one GluN1 gene with diverse splice variants, there are four GluN2 subunits, named GluN2A-D, and two GluN3 subunits [78]. The vast majority of literature on physiologically expressed native NMDA receptors (NMDARs) has concentrated on the subunits GluN2A and GluN2B. During physiological ontogenesis, there is a shift from GluN2B-containing NMDARs in the immature brain towards GluN2A-containing isoforms in adult mammals [79]. This also has functional implications, since GluN2B-containing NMDARs show slower decay times compared with GluN2A-containing receptors, allowing for an increased Ca^2+^ influx in the open state [80]. On the network level, the NMDAR subunit composition governs synaptic plasticity [81,82], and GluN2B-containing receptors predominantly expressed at extrasynaptic sites can laterally diffuse to modulate synaptic plasticity [83]. Interestingly, the developmental shift from GluN2B to GluN2A seems to be reversed in chronically epileptic tissues [84,85]. Intriguingly, recent data showed a conversion of the upregulated GluN2B tyrosine(1472) phosphorylation in the pilocarpine model of chronic temporal lobe epilepsy by the AMPAR antagonists PER and GYKI 52,466 in responders, but not non-responders [86]. This effect has been attributed to increased Pten expression and decreased activity of the Src family-casein kinase 2 signaling pathway [86]. Human astrocytes express NMDARs, including GluN2B [87,88], and PTEN deficiency in the human glioblastoma U87MG cell line was associated with increased proliferation [89], but NMDARs seemed to be less abundant in glioma specimens [90,91]. In particular, GluN1 seemed to be underexpressed [60,61]. Nonetheless, since increased GluN2B serine(1303) phosphorylation was detected in human perigliomal tissue [92], and given that PER is quite effective in suppressing glioma-associated seizures (Table 1) [93,94,95,96,97,98], whether the efficacy of PER in brain tumors could partially rely on PTEN-mediated reversal of upregulated GluN2B tyrosine(1472) phosphorylation is an attractive hypothesis.

The atypical amino acid D-serine was identified as a substitute ligand at the till-then called strychnine-insensitive glycine-binding site of NMDARs [99] and subsequently detected in the cerebral cortex, representing 20–30% of total brain serine [100]. D-serine is produced from L-serine by the glial enzyme serine racemase [101], and the uneven distribution of D-serine in the brain [100] has been found to be reciprocal to the expression of its degrading enzyme D-amino acid oxidase (DAAO) described two decades earlier [102]. In turn, D-serine uptake into glial cells is due to the alanine-serine-cysteine amino acid transporter-2 (ASCT2) [103,104]. For more than 20 years, D-serine has been accepted as the endogenous coagonist of NMDARs [105,106]. With respect to gliomal D-serine metabolism, many groups have studied glioma cell lines, using them as surrogates for forebrain astrocytes. While DAAO appears to be underexpressed in C6 [107] and U87MG glioma cells [108], there is an important interaction between D-serine and nitric oxide (NO), which has been found in U87MG cells: NO suppressed D-serine abundance due to both serine racemase inhibition [109] and DAAO activation [110]. Together with the established upregulation of inducible NO synthase (iNOS) in human glioblastoma specimens [111], this may point to a global D-serine downregulation in glioma tissues. One plausible explanation for overall D-serine suppression in glioma tissues might be the potential cytotoxic effects of DAAO-mediated D-serine degradation leading to H_2_O_2_ production, which could be demonstrated to exert therapeutic relevance in C6 cells [112].

Taken together, GluN2B-mediated mechanisms might be related to epileptogenesis and, hence, specific antagonists against GluN2B-containing NMDARs could be effective in reducing glioma-associated epilepsy. In contrast, D-serine abundance appears to be downregulated in glioma tissues and therefore might be an anti-glioma strategy along with iNOS inhibition.

### 2.2. Metabotropic Glutamate Receptors

Metabotropic glutamate receptors (mGluR) are a family of G-protein-coupled receptors that, with respect to the central nervous system, are predominantly expressed in synapses [113]. A total of eight members (mGluR1-8) are known, which are divided into three groups based on sequence homology, pharmacological properties and intracellular signaling pathways (Figure 2, lower row). Upon the binding of glutamate, the receptors form dimers that are mostly homomers, but there is also evidence that heterodimers exist [114,115].

Group I mGluRs are coupled to G_q_/G_11_. After receptor activation and G-protein dissociation, phospholipase C (PLC) hydrolyzes phosphatidylinositol-4,5-bisphosphate (PIP_2_) to diacylglycerol (DAG) and inositol-1,4,5-trisphosphate (IP_3_), leading to IP_3_-receptor-mediated Ca^2+^ release from the endoplasmatic reticulum. Finally, protein kinase C (PKC) is activated and may phosphorylate downstream targets. Both group Ⅱ and group Ⅲ receptors couple to G_i/o_, and upon receptor activation, lyase activity of adenylate cyclase is attenuated. One major downstream pathway is the cyclic adenosine monophosphate (cAMP)-dependent activation/inactivation of protein kinase A (PKA). Both G_i/o_- and G_q_/G_11_-coupled receptors are linked to major signaling pathways like PI3K/AKT and MAPK, which are essential to the regulation of cell survival and proliferation [116].

Analyses of human tissue freshly taken from surgery and data of permanent lineages show that glioblastoma cells express all types of metabotropic glutamate receptors [60,61,117], with mGluR3 demonstrating the highest expression on average [117]. Hence, it is not surprising that most studies have focused on group 2 receptors.

#### 2.2.1. Group I

Group I consists of mGluR1 and mGluR5. Little is known about the possible role of group I metabotropic receptors in tumorigeneses and glioblastoma progression. Studies employing permanent lineages could show that antagonists of mGluR1 mediated inhibitory effects with respect to cell proliferation and viability in vitro [118,119]. Accompanying immunoblot analysis displayed an overall lower phosphorylation of kinases of the PI3K/AKT pathway. In line with this finding, growth of U87MG xenografts in immunodeficient mice was also attenuated by mGluR1 antagonists [118]. Under hypoxic conditions, inhibition of mGluR5 contributed to an increase of cell death concomitant with a lower AKT phosphorylation [120].

#### 2.2.2. Group II

The second group is formed by mGluR2 and mGluR3. A low expression of mGluR3 in surgery samples was correlated with an overall longer survival after diagnosis [117,121]. Cell culture studies employing primary glioblastoma cells and the U87MG cell line suggested that cell proliferation, survival and migration were dependent on functional mGluR2/3 proteins [122,123,124]. The antitumoral effects were confirmed in nude mice with subcutaneously localized glioma xenografts [122] or orthotopically-implanted tumors [125]. Inhibition of mGluR2/3 signaling may also increase susceptibility to an additional treatment with EGFR antagonist gefitinib in vitro [124].

Furthermore, low mGluR3 expression in patients suffering from glioblastoma is also associated with a higher response to treatment with TMZ [121]. This is consistent with the finding that patients with low mGluR3 and MGMT promotor methylation have the longest survival. Notably, no effect of GluR2/3 inhibition on sensitivity to TMZ was observed in permanent glioblastoma cells, whereas glioma stem-like cells were prone to combined treatment [117]. Unfortunately, experiments of xenografts with patient-derived glioma stem-like cells based on these findings showed only limited effects of GluR2/3 inhibitors on tumor size and no impact on survival at all [117].

#### 2.2.3. Group III

Group Ⅲ consists of mGluR4, mGluR6, mGluR7, and mGluR8. Like group I, our knowledge of their possible function in the context of glioblastoma is scarce. Perturbation of mGluR4-mediated signaling may affect tumor cell growth [126]. The authors showed that activation of mGluR4 by selective agonist VU0155041 reduced proliferation and promoted apoptosis in the LN229 glioblastoma cell line in vitro. These data are in line with the effects of N-phenyl-7-(hydroxyimino) cyclopropa[b]chromen-1a-carboxamide (PHCCC) in medulloblastoma cell lines D283 Med, D341 Med, and DAOY [127]. Activation of mGluR4 by PHCCC reduced tumor cell growth in vitro and in immunodeficient mice [127]. A similar role could be played by mGluR8, as this receptor was identified to suppress glioblastoma cell growth and mediate susceptibility to chemotherapeutics in vitro [128].

To the best of our knowledge, no biological data has been published so far for mGluR6 and mGluR7. The expression of mGluR6, best known for its function in the ON-bipolar cells of the retina, was found to be associated with high-grade pediatric CNS tumors [61].

### 2.3. Neurogliomal Synapse

Since glutamate receptors are expressed on glioblastoma cells, the question of how glutamate may reach these cells was until recently believed to be purely spillover transmission from the same or neighboring glioma cells (autocrine and paracrine transmission). This scenario was dramatically extended by the ultrastructural discovery of synaptic contacts between neurons and glioma cells, referred to as neurogliomal synapses [66,67]. Whether all glutamate receptors expressed by glioma cells (i.e., postsynaptic in nature), as outlined in Section 2.1. and Section 2.2., mediate glutamate effects from these neurogliomal synapses (Figure 1) is an open question, but inhibitors of Ca^2+^-permeable AMPARs may yield intriguing candidates to interfere with glutamatergic transmission at these synapses [67].

### 2.4. Therapeutic Strategies

The current oncological treatment of glioblastoma patients has been excellently reviewed elsewhere [129]. Although temozolomide might exert some anticonvulsant effects [130], here we will focus on pharmacological means of addressing glioma-associated seizures since there is an increasing understanding of the shared mechanisms of tumor progression and epileptogenicity, suggesting that certain compounds could establish novel strategies for the treatment of glioblastoma patients presenting epileptic seizures.

#### 2.4.1. Sulfasalazin

Sulfasalazin (SAS) is an inhibitor of the glutamate-cystine exchanger xCT and was therefore regarded as a potential anti-tumor drug, counteracting glutamate release by glioma cells [34,131]. Unfortunately, however, a human clinical trial adding SAS to radiochemotherapy revealed prolonged seizure-free survival in SAS-coadministered patients, but SAS was associated with hematologic side effects [132]. The multi-kinase inhibitor sorafenib was tested in glioblastoma therapy with limited success [133,134,135,136]. Nonetheless, the effects of sorafenib have to be reconciled since this compound was also identified as an xCT inhibitor [137,138].

#### 2.4.2. Anticonvulsants

The initial enthusiastic observation of a survival benefit in glioblastoma patients treated with adjunctive valproic acid (VPA) to standard radiochemotherapy [139,140,141,142,143] was not corroborated in grade Ⅱ/Ⅱ glioma patients [144] and was then shown from a new perspective by a recent meta-analysis, raising concerns in comparing historical cohorts [145]. On the pathophysiological level, the VPA effect has been attributed to the inhibition of histone deacetylase [146], but again, this cell culture-based in vitro finding was not confirmed in patient-derived glioma tissue specimens [147] or patient-derived low-passage cell lines [148].

Despite there being no specific guidelines for the anticonvulsant choice regarding brain tumor-associated seizures, there is ongoing interest in identifying compounds with anti-tumoral in vitro effects such as levetiracetam (LEV) [149]; see [148]. Among more contemporary anticonvulsants, most patients receive LEV for glioma-associated epileptic seizures, and some recent studies have observed a benefit in overall survival [150,151,152]. Although the mechanism of this survival benefit has not been directly addressed, it may partly be due to a reduced glutamate-to-GABA ratio [153,154].

Although topiramate (TPM) was also considered as the anti-seizure medication of choice in the past [155], its use has been almost abandoned due to side effects and the introduction of LEV. However, since the KAR inhibitor TPM may have anti-proliferative effects in glioblastoma cells [156], it is probably worth reconsidering in patients with insufficient seizure control by LEV and VPA.

#### 2.4.3. Talampanel

Talampanel is an orally bioavailable, noncompetitive antagonist of the AMPA subtype of ionotropic glutamate receptors. In a phase Ⅱ trial [157], it was added to radiotherapy (30-times 0.2 Gy) and concomitant TMZ (75 mg/m^2^ for 6 weeks), subsequently followed by adjuvant TMZ (150-200 mg/m^2^ for 5 days every 28 days for 6 months). In a sub-cohort of 60 patients age-matched to the European Organisation for Research and Treatment of Cancer (EORTC) population, the median survival was significantly prolonged, suggesting that pharmacological inhibition of AMPA receptors could be regarded as a promising therapeutic strategy [157]. However, this effect was only seen in combination with radiotherapy and TMZ, whereas talampanel alone had no significant effect on tumor progression or survival [158].

#### 2.4.4. Perampanel

Perampanel is a structural analogue of talampanel and has been approved as an add-on-anticonvulsant for focal and generalized epilepsy. The encouraging data on talampanel have fostered studies with PER in glioma-associated epilepsy, and since the description of PER administered to a patient with IDH1-wildtype and MGMT-unmethylated glioblastoma who became seizure-free and survived for 18 months [159], a number of rather small studies have explored PER efficacy in glioma-associated seizures (Table 1). Taken together, the 81 patients enrolled, of whom 46 became seizure-free with PER (57%), had a responder rate (i.e., all patients with at least 50% seizure reduction) of 86%. Given the fact that PER was added to a so-far insufficient anti-seizure medication, PER appears to be substantially effective in these patients with glioma-associated epilepsy.

#### 2.4.5. Memantine

The NMDAR inhibitor memantine has been found to inhibit proliferation in GBM cell lines in vitro [160] and in vivo [31]. Currently, patients are being recruited in clinical trials investigating PER, SAS and memantine (Table 2).

## 3. Preclinical Models to Study Glutamate Interaction and Tumor-Associated Epilepsy

A variety of preclinical glioma models exist, ranging from cell cultures to in vivo models employing animal and human tumor cells. Experimental models that focus primary on tumors were recently critically discussed in a review by Lenting et al., 2017 [161]. In this section, we focus on models that are appropriated to investigate the role of glutamate.

### 3.1. Cell Culture Models

Cell culture studies have the decisive disadvantage that the interaction between the microenvironment and the immune system is neglected in principle (Table 3). Moreover, in cell lines like U87MG and U251, which were established several decades ago, genetic changes due to long-term cultivation in serum-containing conditions are likely [162,163]. Nevertheless, most basic mechanistic relationships are based on studies employing these permanent glioma cells (see previous section).

A superior alternative to these permanent cells is patient-derived cell lines in low passages. Published biobanks reflect all kinds of molecular subtypes of glioblastoma [174,175,176,177]. If the molecular status of the primary tumor is known and the corresponding clinical patient data are available, the correspondingly derived cell lines are of particular value for preclinical studies. Employing low-passage cell lines could be used to investigate drug effects on glutamatergic mechanisms in glioblastoma [117,148].

In vitro, glioblastoma cells can also be cultivated as 3D spheroids or organoids [165,166,167,178]. As in 2D cultures, the tumor-surrounding microenvironment is absent. Co-culture models and approaches with limited immune components exist, but these may only reflect selected components and are no full replacement for in vivo conditions [166]. With respect to glutamate-driven processes in glioblastoma, the main focus with these models is on drug screening and as a source for injection in in vivo models or in organotypic brain slices.

### 3.2. Organotypic Brain Slice Cultures

With respect to the 3R principle (replacement, reduction, and refinement), organotypic brain slices can be a valuable link between cell culture studies (2D/3D) and animal experiments. Organotypic brain slice cultures are primarily used to study glioblastoma invasion and angiogenesis [179,180,181,182]. Interestingly, Savaskan et al. (2011) found no direct effect on glioma cells by AMPA receptor inhibition in vitro [168]. However, in the same study on organotypic slices harboring glioma cells, tumor growth was attenuated, and neuronal cell death was alleviated, which highlights the close interaction of glioma cells and peritumoral tissue [168]. Organotypic brain slice cultures may also be used to functionally characterize synaptic interactions, which will gain additional importance with the recent discovery of neurogliomal synapses (see Section 2.3.) [66,67].

**Figure 3:**
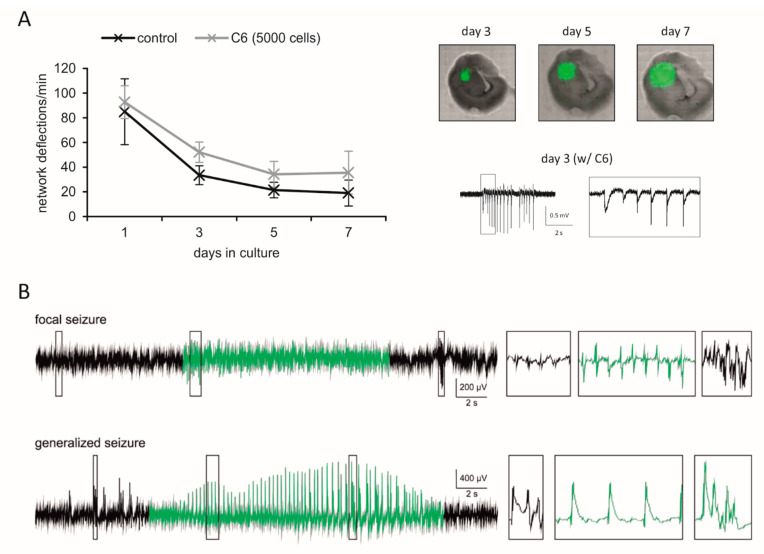
Experimental models to study glutamatergic mechanisms in glioblastoma and epilepsy. (**A**) Coronal slices (350 µm) of 6–8 days-old Fischer rats were prepared to establish organotypic brain slices. The slices were cultured in 6-wells onto Millicell, cell culture inserts (0.4 µm pore size) in slice culture medium (composed of 49% MEM with GlutaMAX, 1% penicillin/streptomycin, 12.5% BME, 12% FCS, and 0.5% glucose). C6 glioma cells (5 × 10^3^ cells) with stable expression of GFP (humanized Renilla reniformis green fluorescence protein) were placed onto the slices in cortical areas. For electrophysiological recordings, slices were exposed to artificial cerebrospinal fluid (aCSF) solution [164] without Mg^2+^, 8 mM KCl and 5 µM gabazine and network deflections were quantified in the last 20 min after two hours of perfusion. Data represent mean of 3–4 separate experiments ± SEM. At the same day, C6 glioma cell growth was estimated by laserscanning microscopy. (**B**) Orthotopically-growing F98 glioma in male Fischer rats exhibits an epileptiform phenotype. Therefore, 1 × 10^5^ F98 glioma cells were injected in the frontal cortex of Fischer rats (for experimental details see [169]). Additionally, two electrodes were placed epidurally above the cortex, and continuous 24/7 video-EEG data (sample rate 500 Hz, low-pass filter 30 Hz) were recorded. The F98 model exhibit seizures (green traces) and interictal events (black traces). Representative parts of the EEG trace are enlarged on the right-hand side (boxes with 10-fold time resolution but identical amplitude scaling). Consistent with the orthotopic inoculation of F98 cells, both focal (upper row) and secondarily generalized seizures (lower row) can be detected.

Here, we show that C6-bearing slices based on brain tissue from 6–8 days-old Fischer rats with C6 cells also represent a feasible tool to investigate the network activity (Figure 3A). A high synaptic synchrony of cortical neurons that declines as the incubation progresses was estimated, which was expected for slice cultures without glioma [183].

### 3.3. In Vivo Models

Only models with orthotopic glioma are useful models to investigate glioma-associated epilepsy. In order to reliably verify tumor-associated seizures, EEG recordings are required, supported by video recordings. To the best of our knowledge, only a small number of in vivo rodent models with glioma-associated epilepsy are published so far [184].

A well-established in vivo model is the implantation of orthotopically-growing F98 rat glioma cells in Fischer rats [185]. Depending on the number of cells injected, the animals survive for about 2–5 weeks after glioma implantation [169,185,186,187]. Recently, the model was reported to develop a tumor-associated seizure during glioma progression [169,170]. In line with own data from video-EEG analyses (Figure 3B), Bouchaert et al. (2020) observed seizures in all tumor-bearing animals in their study. However, the model shows a heterogeneous distribution of the seizures, with some animals suffering from more seizures per day and others having only a few seizures over the entire period of the experiment [169,170]. Between the seizures, interictal events can be found in the EEG (black traces in Figure 3B). These spikes become more frequent as tumor growth is proceeding. Interestingly, we found a decrease in spike rates during the pre-moribund state prior sacrificing the animals, presumably due to neuronal death, increasing intracranial pressure and the loss of network integrity (data not shown).

C6 glioma cells have been used in a variety of experimental studies in the past [188]. Like the F98 model, the glioma-based epileptiform activity of C6 glioma is verified [171,172]. Following external auditory stimuli, the authors recorded seizures in EEG analyses. Additionally, spontaneous seizures could be documented in our video EEG recordings, although these are less frequent compared with the F98 glioma model (data not shown). In brain slices with C6 tumors, field potential records revealed that epileptiform discharges are glutamatergic [164].

Recently, Hatcher et al. (2020) published a CRISPR-based mice model [58]. Fetal deletion of *Pten*, *Nf1*, and *Trp53* leads to an onset of glioblastoma with an epileptiform phenotype. Like the rat model of F98 glioma, abnormalities become visible in the EEG in the mice model, and from P60, seizures are present. Another mouse model with a glioma-associated epileptiform phenotype is also based on *Pten* and *TrP53* deletions [173]. In the late stages of tumor progression, the animals exhibit interictal events and seizures. Both models combine the great advantage that the tumors are based on alterations of human glioblastomas.

In addition to immunocompetent models of rodent glioma, primary human glioblastoma cells could be used in nude mice. By implanting primary human glioblastoma cells (GBM12 or GBM22), Buckingham and colleagues recorded epileptiform events in tumor-bearing C.B-17 SCID mice [34]. The advantage of using human glioblastoma cells in immunodeficient animals compared with the immunocompetent rodent models of glioma must be weighed up according to the scientific question.

## 4. Conclusions and Future Perspectives

A number of glutamatergic mechanisms have been revealed to be involved in both glioma progression and glioma-associated epilepsy, ultimately culminating in the discovery of the neurogliomal synapse. Hence, pharmacological interventions in these mechanisms are promising strategies in addressing both tumor progression and epilepsy. Findings on AMPAR antagonists and glutamate transporter inhibitors have revealed potentially beneficial effects in a variety of glioma models, but human studies are rare, and being easily underpowered, which is an inherent challenge. However, a number of players in this glutamatergic interplay, such as metabotropic glutamate receptors and D-serine, have only been marginally addressed so far and certainly merit more attention in future studies. Thus, it is conceivable that combined interventional strategies rather than individual clinical proving may be helpful in promoting new therapies [189].

In the absence of glioma-specific guidelines for treating symptomatic seizures, glioma-associated epilepsy was controlled with LEV and VPA [25,190], but further compounds were also effective [191]. The AMPAR antagonist PER may offer the opportunity to address the two faces of glutamatergic effects in glioma, i.e., progression and epileptogenicity. Currently, there are further ongoing clinical trials evaluating PER efficacy and, in addition, brivaracetam and lacosamide are increasingly applied due to their parenteral formulations. Since the latter two are assumed to reduce glutamate release not only from neurons, but also from astroglia [192,193], the glutamatergic interplay in the glioma-surrounding network will reasonably attract more interest in future studies.

## Figures and Tables

**Table 1:** Studies with perampanel add-on therapy for glioma-associated seizures.

Reference	Patients Enrolled	Perampanel Therapy	Seizure Reponse
Vecht et al., 2017 [93]	12 patients	2–12 mg/d	seizure-free = 6/12
	9 male, 3 female	follow-up = 6 months	≥50% reduction = 3/12
	median = 41 years		responder rate = 75%
Dunn-Pirio et al., 2018 [94]	8 patients	2–8 mg/d	seizure-free = 5/8
	6 male, 2 female	follow-up = 16 weeks	≥50% reduction = 1/8
	median = 45 years		responder rate = 75%
Izumoto et al., 2018 [95]	10 patients	4–8 mg/d	seizure-free = 6/10
	6 male, 4 female	follow-up = 6 months	≥50% reduction = 4/10
	median = 59 years		responder rate = 100%
Maschio et al., 2019 [96]	11 patients	7.3 mg/d	seizure-free = 5/12
	9 male, 2 female	follow-up = 12 months	≥50% reduction = 4/12
	median = 54 years		responder rate = 82%
Chonan et al., 2020 [97]	18 patients	2–4 mg/d	seizure-free = 17/18
	9 male, 9 female	follow-up = 10.6 months	≥50% reduction = 0/18
	median = 49 years		responder rate = 94%
Coppola et al., 2020 ^1^ [98]	36 patients	2–12 mg/d	seizure-free = 7/21
	23 male, 13 female	follow-up = 12 months	≥50% reduction = 12/21
	median = 46 years		responder rate = 90%

^1^ Patient data from intention-to-treat analysis, seizure response data from per-protocol analysis.

**Table 2:** Studies with add-on drugs for glioma-associated seizures.

Title (Trial)	Status	Interventions	Location
Perampanel for the reduction of seizure frequency in patients with high-grade glioma and focal epilepsy (NCT04650204)	Not yet recruiting	Perampanel	Jacksonville, FL, USA
Effect of perampanel on peritumoral hyperexcitability in HGG (NCT04497142)	Recruiting	Perampanel	Boston, MA, USA
Sulfasalazine and stereotactic radiosurgery for recurrent glioblastoma (NCT04205357)	Recruiting	Sulfasalazine	Bergen, Norway
Efficacy and Safety of perampanel in combination in glioma-refractory epilepsy (NCT03636958)	Recruiting	Perampanel	Marseille, France
Memantine for prevention of cognitive late effects in pediatric patients receiving cranial radiation therapy for localized brain tumors (NCT03194906)	Recruiting	Memantine	Memphis, TN, USA
Temozolomide, memantine hydrochloride, mefloquine, and metformin hydrochloride in treating patients with glioblastoma multiforme after radiation therapy (NCT01430351)	Active, not recruiting	Memantine, mefloquine, metformin	Houston, TX, USA

**Table 3:** Relevant Preclinical Models to Study Glutamatergic Mechanisms in Glioma and Tumor-Associated Epilepsy.

Level	Model	Glioma	Advantages/Disadvantages
in vitro	permanent cell lines	rodent and human cells [45,128,164]	(+) high throughput
			(−) genetic drift
			(−) no microenvironment
	patient-derived cell lines	human (primary) glioblastoma cells [117,148]	(+) high throughput (+) genetic status of primary tumor and clinical data accessible (−) no microenvironment
	spheroids/organoids	human and rodent glioblastoma [165,166,167]	(+) median throughput (−) no microenvironment
ex vivo	organotypic slice cultures with glio- blastoma cells	human or rodent glioblastoma [168]	(+) median throughput (+) genetic manipulation feasible (+) interaction with healthy brain tissue
			(−) only short-time monitoring (1-3 weeks)
			(−) microenvironment lacking immune system
			(−) animal consuming research
in vivo ^1^	orthotopic rat glioma	F98 and C6 rat [169,170,171,172]	(+) glioma-associated seizures (+) immunocompetent (−) ethical issues related to animal studies (−) low throughput (−) no genetic variances
	orthotopic mice glioma	murine glioma [58,173]	(+) glioma-associated seizures (+) immunocompetent (+) genetic alterations based on human glioma (−) ethical issues related to animal studies (−) low throughput (−) low genetic variances
	orthotopic human glioblastoma	GBM12/GBM22 [34]	(+) glioma-associated seizures (−) ethical issues related to animal studies (−)low throughput (−) immunodeficient host (−) no genetic variances

^1^ only models with documented epileptic seizures are included.

## Data Availability

Not applicable

## Abbreviations

aCSF	artificial cerebrospinal fluid
AKT	protein kinase B
AMPA	α-amino-3-hydroxy-5-methyl-4-isoxazolepropionic acid
AMPAR	AMPA receptor
ASCT2	alanine-serine-cysteine amino acid transporter-2
BCAT1	branched-chain amino acid transaminase 1
cAMP	cyclic adenosine monophosphate
CDKN2A/B	cyclin dependent kinase inhibitor 2A/B
CDK4	cyclin-dependent kinase 4
DAAO	D-amino acid oxidase
DAG	diacylglycerol
D-2HG	D-2-hydroxyglutarate
EAAT2	excitatory amino acid transporter 2
GLT-1	glutamate transporter 1
IDH1	isocitrate dehydrogenase 1
iNOS	inducible NO synthase
IP_3_	inositol-1,4,5-trisphosphate
KAR	kainate receptor
LEV	levetiracetam
MAPK	mitogen-activated protein kinase
MGMT	O^6^-methylguanine-DNA methyltransferase
mGluR	metabotropic glutamate receptor
Nf1	neurofibromin 1
NMDA	N-methyl-D-aspartate
NMDAR	NMDA receptor
PDGFRA	platelet-derived growth factor receptor A
PER	perampanel
PI3K	phosphatidylinositol-3-kinase
PKA	protein kinase A
PLC	phospholipase C
PTEN	phosphatase and tensin homolog
SAS	sulfasalazine
SV2A	synaptic vesicle glycoprotein 2A
TMZ	temozolomide
TPM	topiramate
TP53	tumor protein P53
VPA	valproic acid
xCT	solute carrier family 7 member 11
